# Androgen receptors in areas of the spinal cord and brainstem: A study in adult male cats

**DOI:** 10.1111/joa.13407

**Published:** 2021-02-22

**Authors:** Rosa L. Coolen, Jacqueline C. Cambier, Panagiota I. Spantidea, Els van Asselt, Bertil F. M. Blok

**Affiliations:** ^1^ Department of Urology Erasmus Medical Center Rotterdam The Netherlands

**Keywords:** androgen, autonomic function, medulla oblongata, mesencephalon, PG21, spinal cord

## Abstract

Sex hormones, including androgens and estrogens, play an important role in autonomic, reproductive and sexual behavior. The areas that are important in these behaviors lie within the spinal cord and brainstem. Relevant dysfunctional behavior in patients with altered androgen availability or androgen receptor sensitivity might be explained by the distribution of androgens and their receptors in the central nervous system. We hypothesize that autonomic dysfunction is correlated with the androgen sensitivity of spinal cord and brainstem areas responsible for autonomic functions. In this study, androgen receptor immunoreactive (AR‐IR) nuclei in the spinal cord and brainstem were studied using the androgen receptor antibody PG21 in four uncastrated young adult male cats. A dense distribution of AR‐IR nuclei was detected in the superior layers of the dorsal horn, including lamina I. Intensely stained nuclei, but less densely distributed, were found in lamina X and preganglionic sympathetic and parasympathetic cells of the intermediolateral cell column. Areas in the caudal brainstem showing a high density of AR‐IR nuclei included the area postrema, the dorsal motor vagus nucleus and the retrotrapezoid nucleus. More cranially, the central linear nucleus in the pons contained a dense distribution of AR‐IR nuclei. The mesencephalic periaqueductal gray (PAG) showed a dense distribution of AR‐IR nuclei apart from the most central part of the PAG directly adjacent to the ependymal lining. Other areas in the mesencephalon with a dense distribution of AR‐IR nuclei were the dorsal raphe nucleus, the retrorubral nucleus, the substantia nigra and the ventral tegmental area of Tsai. It is concluded that AR‐IR nuclei are located in specific areas of the central nervous system that are involved in the control of sensory function and autonomic behavior. Furthermore, damage of these AR‐IR areas might explain related dysfunction in humans.

## Graphical abstract

In this study, androgen receptor immunoreactive (AR‐IR) nuclei in the spinal cord and brainstem were studied using the androgen receptor antibody PG21 in four uncastrated young adult male cats. It is concluded that AR‐IR nuclei are located in specific areas of the central nervous system that are involved in the control of sensory function and autonomic behavior. Furthermore, damage of these AR‐IR areas might explain related dysfunction in humans.

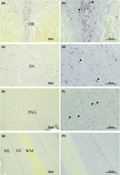

## 1 INTRODUCTION

Androgens play an important role in autonomic, reproductive, and sexual behavior (Cunningham et al., 2012; Santi et al., 2018). The central nervous system areas that are important in these behaviors lie within the spinal cord and brainstem. Relevant dysfunctional behavior in patients can be due to an altered availability of androgens in the central nervous system. This can be caused by androgen deprivation therapy, androgen insensitivity syndrome, hypogonadism, Kennedy's disease, and many other causes. Androgen deprivation therapy is one of the standard treatments of metastasized prostate cancer and may cause cognitive dysfunction, including dementia, impotence, and other side effects (Donovan et al., 2018; McGinty et al., 2014; Nead et al., 2017). Androgen insensitivity syndrome and Kennedy's disease are caused by androgen receptor mutations which alter the structure or function of the receptor (Brinkmann et al., 1995). Hypogonadism is a disorder that results in a decreased production of androgens. It is diagnosed according to the guideline of the European Association of Urology when signs and symptoms of androgen deficiency occur together with consistently low serum testosterone levels (Dohle et al., 2019). One of such symptoms is erectile dysfunction, which is associated with decreased levels of free testosterone (Huang et al., 2019). The consequences of reduced testosterone in the central nervous system are claimed to be sexual dysfunction, such as impotence, and impaired cognition (Bravo et al., 2017; Kawano et al., 2003; Ophoff et al., 2009; Rana et al., 2011; Takov et al., 2018; Traish et al., 2015). On the contrary, increased levels of testosterone are associated with altered behavioral responses such as aggression (Carré & Archer, 2018). To date, the pathophysiology of autonomic dysfunction associated with an altered level of androgens or an altered sensitivity/expression of androgen receptors is not well‐known. We hypothesize that autonomic dysfunction in patients with altered levels of androgens is correlated with the androgen sensitivity of spinal cord and brainstem areas that are responsible for autonomic functions. Autonomic reflexes originate in the brainstem. It is, therefore, important to expand the knowledge on the androgen receptor distribution within these areas of the central nervous system.

Androgen receptors are members of a superfamily of ligand‐dependent transcription factors. The androgen receptor genes are located on the X chromosome and expressed in most of the organs in the body (Hunter et al., 2018). They are located in an inactive form in the cytoplasm and are activated as a result of strong affinity‐binding with androgens (mostly testosterone and dihydrotestosterone) and the subsequent formation of the androgen‐androgen receptor complex. The activation of this complex causes translocation to the nucleus, where it binds directly to DNA or through histones or through chromatin remodeling (DNA binding‐dependent). The activated complex can also induce activation of secondary messenger pathways including ERK, Akt and MAPK (non‐DNA binding dependent) to regulate androgen‐regulated genes (Brinkmann et al., 1999; Claessens et al., 2008; Gioeli & Paschal, 2012; Hipkaeo et al., 2004). For an optimal androgen receptor regulation, regulators, directly or indirectly inhibit androgen receptor activation by reducing the concentration of androgens. Those molecules can be cytokines, growth factors or others. O'Bryant and Jordan showed that there are five different putative cofactors which have the potential to participate in motoneuronal responses to androgens (O'Bryant & Jordan, 2005).

It is known from immunohistochemical receptor studies in various species that androgens may have effects on specific regions of the central nervous system. Studies in rats have shown androgen receptor expression in autonomic behavior related structures of the spinal cord and brainstem such as the area postrema, the intermediolateral cell column, the nucleus of the solitary tract, and the periaqueductal gray (PAG) (Sar & Stumpf, 1975; Simerly et al., 1990). In rhesus and cynomolgus macaques, androgen sensitive structures were found in forebrain areas, but the location of androgen receptor immunoreactive (AR‐IR) cells in the spinal cord and brainstem was not investigated in monkeys except for one study that described AR‐IR cells in the dorsal raphe (Bethea et al., 2015; Choate et al., 1998; Clancy et al., 1992; Michael et al., 1989). Thus far, the androgen receptor distribution in the spinal cord and brainstem of mammals other than rodents has not been studied. The location of AR‐IR cells in the spinal cord and brainstem has not been investigated in cats and humans either. The cat nervous system has a great resemblance to that of humans. Therefore, it is an excellent model for studying the central nervous system.

The present study investigates which areas of the spinal cord and brainstem of adult male cats contain AR‐IR nuclei. A comparison is made between the distribution of AR‐IR nuclei in the cat and that previously described in the literature on rodents and monkeys. The possible roles of androgen receptors in specific spinal cord and brainstem areas in autonomic function and dysfunction are discussed. Furthermore, the distribution of androgen receptors in the spinal cord and brainstem is compared to the distribution of estrogen receptors in the same areas as described in earlier studies.

## 2 MATERIALS AND METHODS

### 2.1 Animals

All surgical procedures, pre‐ and postoperative care, handling, and housing of the cats were in accordance with the protocols approved by the Committee on Animal Experiments of the Faculty of Medical Sciences of the University of Groningen. Four uncastrated young adult male cats (12–24 months, Harlan) received 6 ml, 6% sodium pentobarbital intraperitoneally, and were perfused transcardially with Saline in 0.01 M Phosphate Buffer (PB, pH 7.4), followed by 4% paraformaldehyde in 0.1 M PB (pH 7.4). The spinal cords and brainstems were removed and post fixed for 2 h at 4°C.

### 2.2 Immunohistochemistry

Sixty µm sections of the spinal cord and brainstem were cut on a Vibratome in cold Tris buffered saline (TBS, pH 7.4). One of four sections was pretreated with 0.3% H_2_0_2_ for 30 min, blocked with 5% normal donkey serum for 30 min at room temperature, and incubated with the primary antibody against androgen receptors, PG21 (donated by Dr Gail S. Prins, University of Chicago, IL, 1.4 µg/ml) diluted in 1% normal donkey serum at 4°C, for three days (Prins et al., 1991). Subsequently, biotinylated donkey anti‐rabbit immunoglobulin (Jackson Immuno Research, diluted 1:500 with 1% normal donkey serum) was used as secondary antibody. The sections were incubated for one hour at room temperature, followed by incubation in avidin‐biotin‐complex‐peroxidase (Vectastain, Vector, 1:400 in TBS, 1 h at room temperature). Following antibody incubation, the sections were rinsed with TBS overnight. Finally, the sections were incubated with 0.04% 3,3' diaminobenzidine tetrahydrochloride (DAB), 0.2% nickel ammonium sulfate, and 0.01% H_2_0_2_ in TBS for 5 min, resulting in a dark precipitate. The blocking serum, primary and secondary antibody were diluted in 0.05 M Tris buffer, 0.5 M NaCl, and 0.5% Triton X‐100. In order to test the specificity of the AR‐IR labeling, appropriate controls were made in a series of sections by omitting the primary antibody to verify the specificity of the AR‐IR labeling. The specificity was previously shown using the antibody on multiple positive and negative controls and by performing competition studies (Prins et al., 1991). Another series of sections was counterstained with the Nissl staining method (Avendaño & Verdu, 1992). The sections were mounted on gelatin‐covered slides and coverslipped with DePeX (Gurr, BDH laboratory).

### 2.3 Data analysis

**FIGURE 1:**
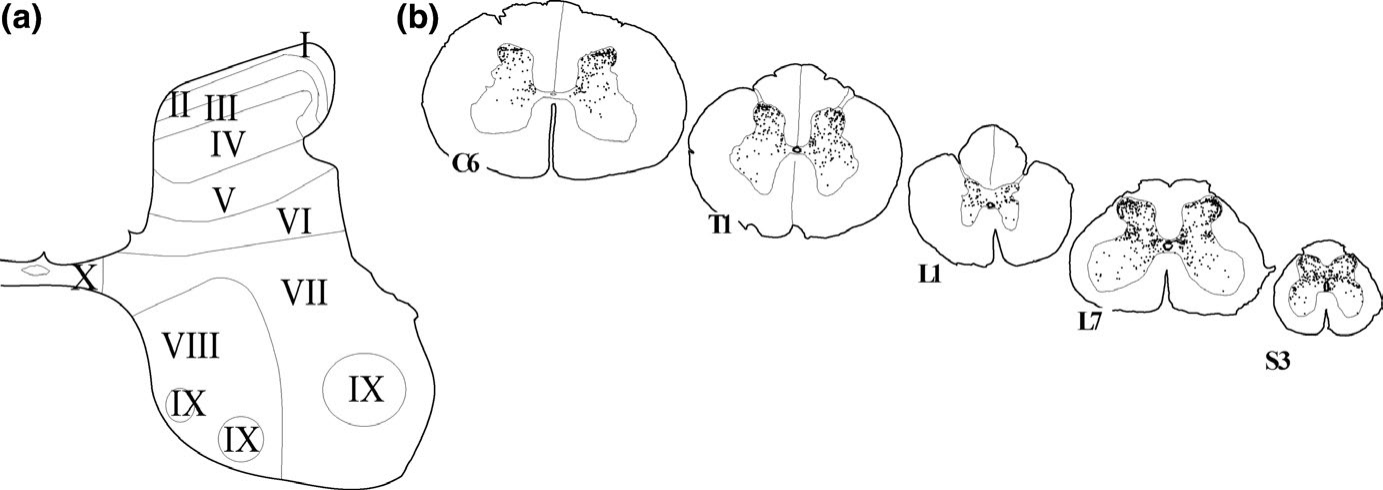
AR‐IR nuclei in the spinal cord. (a) The laminae of Rexed in a schematic drawing of the 6th cervical segment. (b) Spinal cord sections (C6‐S3). Each dot represents one AR‐IR nucleus

AR‐IR nuclei were plotted with a computerized X‐Y stage on a Zeiss Axioplan microscope connected to a PC with the Neurolucida system (MicroBrightField). The drawings of the sections of the spinal cord and brainstem containing plotted nuclei were converted to Encapsulated PostScript (EPS) files and further processed in Adobe Illustrator CC 2018. Example drawings of representative sections at specific levels are presented in the results section (Figures 1 and 3). The results were quantified with a Heidelberg Topaz II + scanner (Kiel, Germany) and Linocolor 5.0 software. The nuclear AR labeling was quantified in a semiquantitative manner. For each area of the spinal cord and the brainstem two aspects of the AR‐IR nuclei were determined: the density of the distribution of AR‐IR nuclei and the intensity of nuclear staining. The distribution of labeled nuclei and the intensity of the labeling are presented in Table 1. A Nikon Ts2‐FL microscope was used to take bright field photomicrographs. Nissl‐stained sections were used to localize the spinal cord nuclei, brainstem nuclei and fiber tracts using the cytoarchitectonic atlas of Berman (Berman, 1968).

**TABLE 1:** Nuclear AR labeling in the spinal cord and brainstem

Region	Distribution of AR‐IR nuclei	Intensity of nuclear AR labeling
Spinal cord		
Central zone (lamina X)	+	+++
Dorsal horn, deep layers	+	+
Dorsal horn, superior layers (I,II,III)	++	+++
Intermediate zone	+	+++
Intermediolateral column	+	+++
Ventral horn	+	+
Medulla oblongata		
Ambiguus nucleus	+	+
Area postrema	++++	++++
Cochlear nuclei	++	+
Cuneate nucleus, caudal division	+	++
Dorsal motor vagus nucleus	+++	++
Gracile nucleus, caudal division	‐	‐
Hypoglossal nucleus	‐	‐
Inferior central raphe nucleus	+	+
Inferior olive (principal nucleus, medial accessory nucleus, dorsal accessory nucleus)	+	+
Inferior vestibular nucleus	+	++
Lateral reticular nucleus	‐	‐
Lateral tegmental field	++	+++
Medial tegmental field	+	+
Nucleus raphe magnus	+	+
Nucleus raphe pallidus	‐	‐
Praeposital hypoglossal nucleus	+	+
Preolivary nucleus	+	++++
Restiform body	‐	‐
Retroambiguus nucleus	+	+++
Retrotrapezoid nucleus	+++	+++
Solitary tract, medial and lateral nucleus	++	++
Spinal trigeminal nucleus	++	++
Superior olive, lateral and medial nucleus	‐	‐
Trapezoid body nucleus	‐	‐
Ventral horn of the caudal brainstem	+	+
Vestibular complex	‐	‐
Pons and cerebellum		
Central linear nucleus of the raphe	+++	+++
Cerebellum	‐	‐
Facial nucleus	‐	‐
Kölliker‐Fuse nucleus	++	++
Motor trigeminal nucleus	+	+
Parabrachial nucleus	++	++
Pontine continence center (L‐region)	‐	‐
Pontine micturition center (M‐region)	‐	‐
Superior central raphe nucleus	‐	‐
Mesencephalon		
Brachial nucleus of the colliculus inferior	+	+
Central tegmental field	++	+++
Dorsal raphe nucleus	++++	+++
Edinger Westphal nucleus	++	+++
Inferior colliculus	‐	‐
Medial pretectal area	++	++
Mesencephalic tegmental field	++	+++
Oculomotor nucleus	+	+
Periaquaductal gray (PAG)	++++	++
Red nucleus	‐	‐
Retrorubral nucleus	+++	+++
Substantia nigra, reticular and compact division	+++	+++
Superior colliculus	++	++
Trochlear nucleus	‐	‐
Ventral tegmental area of Tsai	+++	++

The table shows the density of the distribution of AR‐IR nuclei and the intensity of nuclear AR‐IR in the spinal cord and brainstem. The density and intensity were defined as: low (+), medium (++), high (+++), and very high (++++). The description includes the spinal cord, the medulla oblongata, the pons, the cerebellum, and the mesencephalon. Some regions are located in more than one brainstem region (e.g. trigeminal tract). These regions are only referred to in the table in the brainstem region in which the most prominent labeling was found.

## 3 RESULTS

AR‐IR was predominantly found in the nuclei of neurons, but occasionally also in nuclei of glial and ependymal cells. AR‐IR cells were present throughout the spinal cord and brainstem. The immunostaining varied from light gray to intense black. AR‐IR nuclei in the white matter represented, in all likelihood, glial cells. No staining was found in control sections. Two outcome measures were determined for each spinal cord and brainstem area: the density of the distribution of AR‐IR nuclei and the nuclear intensity of the staining. The density of the distribution of AR‐IR nuclei and the intensity of nuclear staining in areas of the spinal cord and brainstem are presented in Table 1. These results were consistently found in each of the four cats.

### 3.1 Spinal cord

**FIGURE 2:**
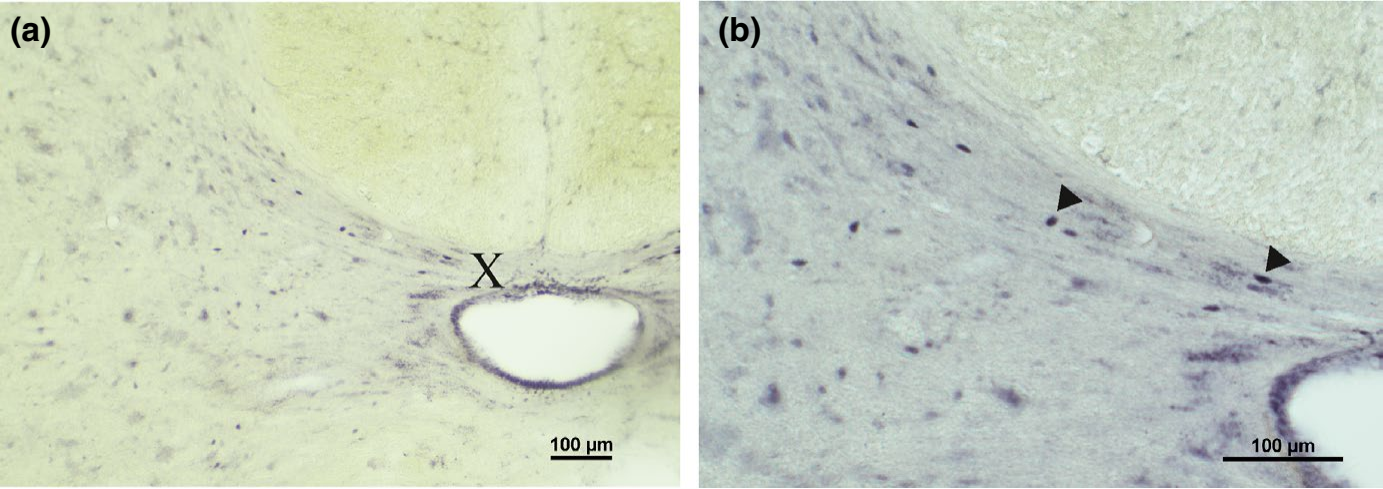
AR‐IR nuclei in the spinal cord. Bright field photomicrographs to illustrate AR‐IR labeling in the spinal cord. Enlargement (a): ×10; (b): ×20. (a) and (b) The intermediate zone, lamina X and the central canal of spinal segment L7. X, laminae of Rexed

The distribution of AR‐IR nuclei was moderate to low in density at every level of the spinal cord (Figures 1 and 2). Most densely labeled were the superior layers (layer I, II, and III) of the dorsal horn and the intermediolateral cell column of the thoracic, lumbar and sacral spinal cord, which contain preganglionic sympathetic and parasympathetic motoneurons. The deep layers of the dorsal horn and the ventral horn were sparsely labeled throughout the spinal cord.

Intensely stained nuclei were observed in the central (lamina X) and intermediate zone at all spinal levels, and in the preganglionic sympathetic and parasympathetic cells of the intermediolateral cell column at thoracic, lumbar, and sacral levels.

### 3.2 Medulla oblongata

**FIGURE 3:**
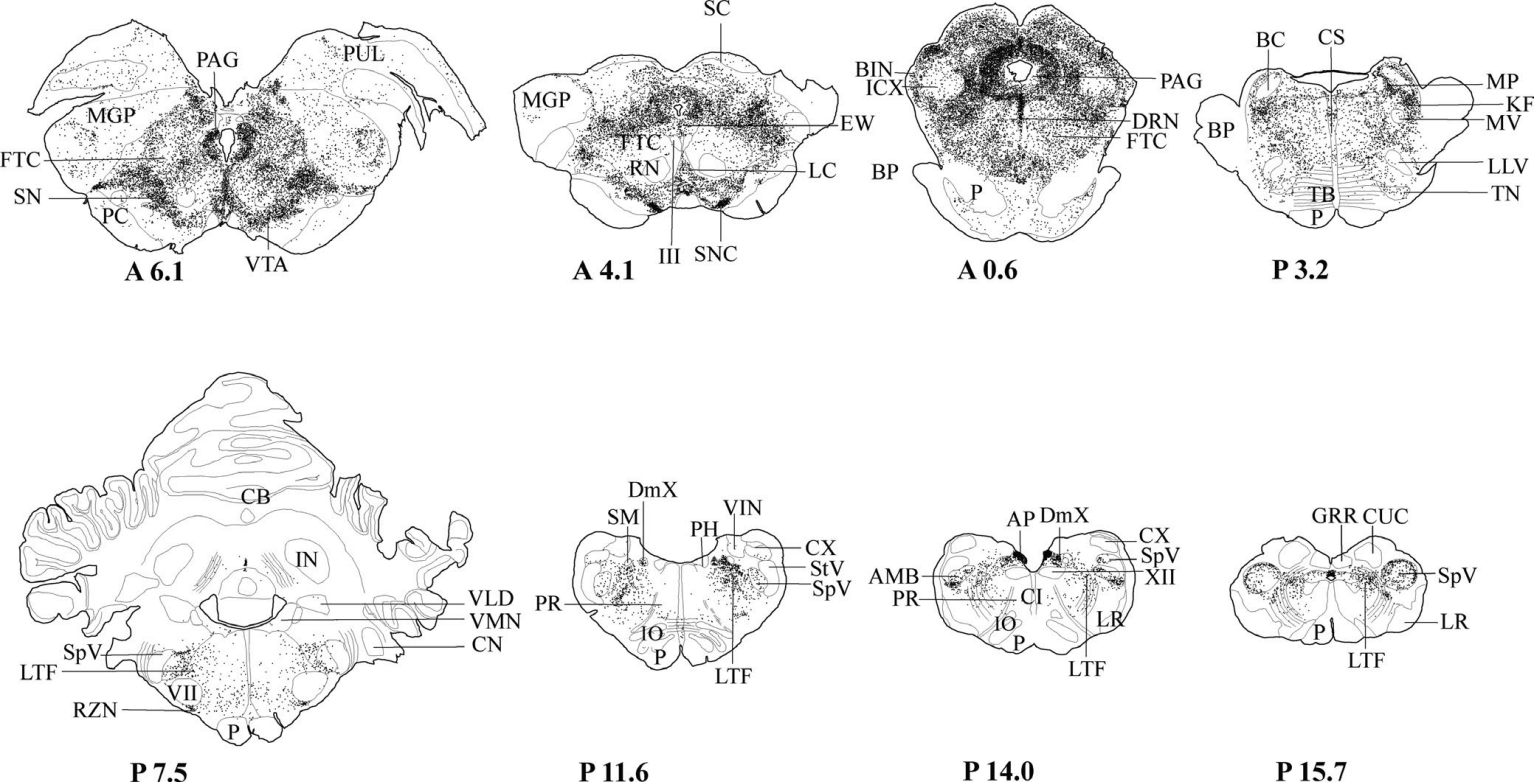
AR‐IR nuclei in the brainstem. Frontal brainstem sections, rostral [A6.1] to caudal [P15.7]. A6.1 corresponds with 6.1 mm anterior (A) and P15.7 corresponds with 15.7 mm posterior (P) to the frontal zero (interaural) plane. Each dot represents one AR‐IR neuron. The coordinates refer to corresponding coordinates in Berman's atlas (Berman, 1968). III, oculomotor nucleus; VII, facial nucleus; XII, hypoglossal nucleus; AMB, ambiguus nucleus; AP, area postrema; BC, brachium conjunctivum; BIN, brachial nucleus of the colliculus inferior; BP, brachium pontis; CB, cerebellum; CI, inferior central nucleus; CN, cochlear nuclei; CS, superior central nucleus; CUC, cuneate nucleus, caudal division; CX, external cuneate nucleus; DmX, dorsal motor vagus nucleus; DRN, dorsal raphe nucleus; EW, Edinger Westphal nucleus; FTC, central tegmental field; GRR, gracile nucleus, rostral division; ICX, external nucleus of the inferior colliculus; IN, nucleus interpositus; IO, inferior olive; KF, Kölliker‐Fuse nucleus; LC, central linear nucleus of the raphe; LLV, ventral nucleus of the lateral lemniscus; LR, lateral reticular nucleus; LTF, lateral tegmental field; MGP, principal nucleus of the medial geniculate body; MP, medial parabrachial nucleus; MV, motor trigeminal nucleus; P, pyramidal tract; PAG, periaqueductal gray; PC, cerebral peduncle; PH, praeposital hypoglossal nucleus; PR, paramedian reticular nucleus; PUL, pulvinar; RN, red nucleus; RZN, retrotrapezoid nucleus; SC, superior colliculus; SM, medial nucleus of the solitary tract; SN, substantia nigra; SNC, substantia nigra compact division; SpV, spinal trigeminal nucleus; StV, spinal trigeminal tract; TB, trapezoid body; TN, nucleus of the trapezoid body; VIN, inferior vestibular nucleus; VLD, lateral vestibular nucleus; VM, medial vestibular nucleus; VTA, ventral tegmental area of Tsai

The distribution of AR‐IR nuclei was dense in the area postrema, the dorsal motor vagus nucleus, and the retrotrapezoid nucleus (Figure 3: P7.5, P14.0). A less dense distribution of AR‐IR nuclei was found in the cochlear nuclei, the lateral tegmental field, the nucleus of the solitary tract, and the spinal trigeminal nucleus (Figure 3: P7.5‐P14.0). AR‐IR nuclei in the spinal trigeminal nucleus were mainly located in the outer layer of the pars caudalis. A sparse distribution of AR‐IR nuclei was found in the ambiguus nucleus, the cuneate nucleus, the inferior central raphe nucleus, the inferior vestibular nucleus, the medial tegmental field, the nucleus raphe magnus, the praepostial hypoglossal nucleus, the preolivary nucleus, the principal, medial and dorsal accessory inferior olive nucleus, the retroambiguus nucleus and the ventral horn of the caudal brainstem (Figure 3: P3.2‐P15.7). The gracile nucleus, the hypoglossal nucleus, the lateral reticular nucleus, the nucleus raphe pallidus, the restiform body, the superior olive, the trapezoid body nucleus, and the vestibular complex did not contain AR‐IR nuclei (Figure 3: P3.2‐P15.7).

A high intensity of nuclear AR‐IR was observed in the area postrema, the lateral tegmental field, the preolivary nucleus, the retroambiguus nucleus, and the retrotrapezoid nucleus.

### 3.3 Pons and cerebellum

**FIGURE 4:**
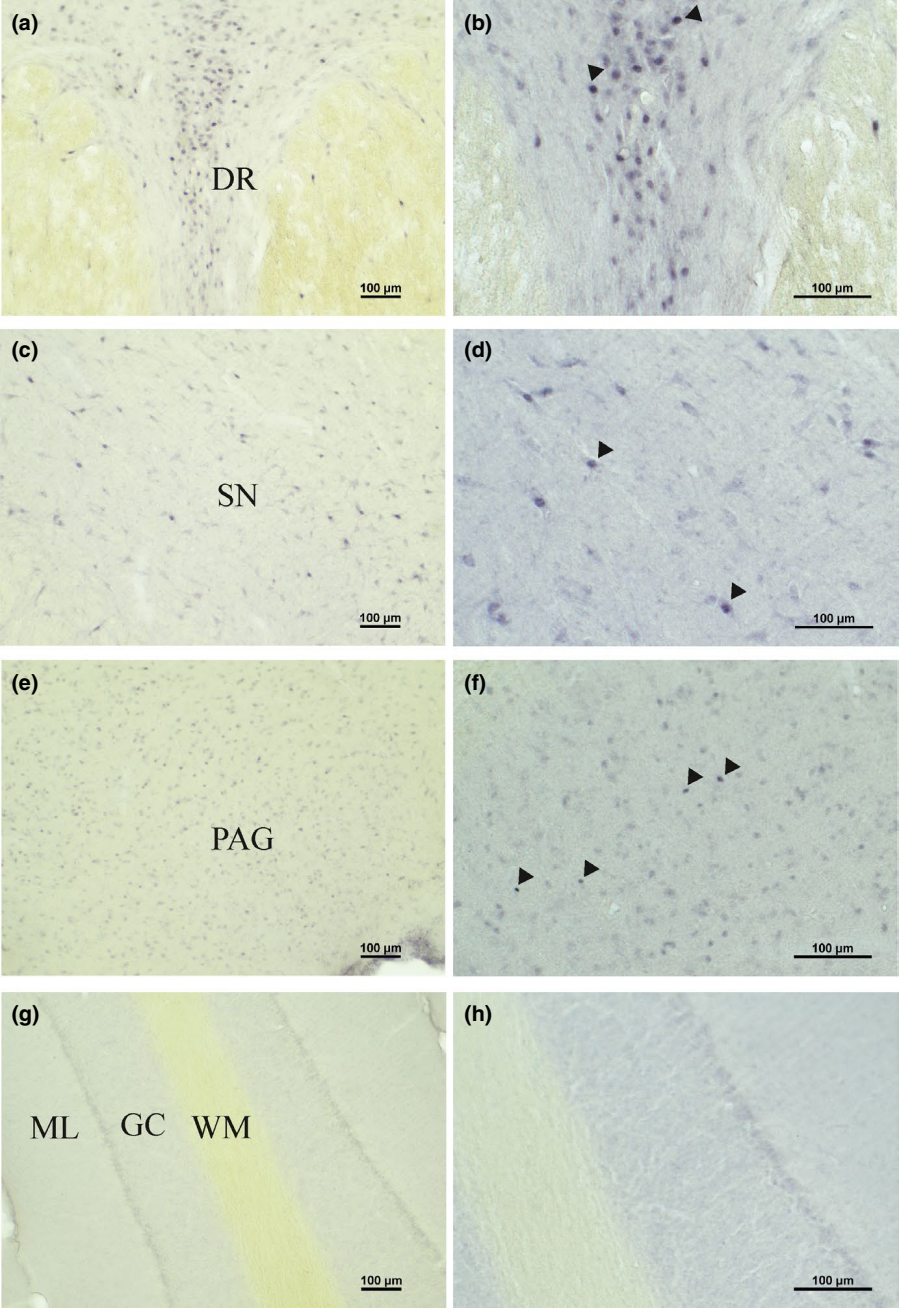
AR‐IR nuclei in the brainstem. Bright field photomicrographs to illustrate AR‐IR nuclei in the brainstem. Enlargement (a, c, e, g): ×10; (b, d, f, h): ×20. (a) AR labeling in the dorsal raphe nucleus. (b) Enlargement of AR‐IR nuclei in the dorsal raphe nucleus. (c) and (d) AR‐IR nuclei in the substantia nigra. (e) and (f) AR‐IR nuclei in the PAG. (g) and (h) Absence of AR‐IR nuclei in the cerebellar cortex. DR, dorsal raphe nucleus; GC, granule cell layer; ML, molecular layer; PAG, periaqueductal gray; SN, substantia nigra; WM, white matter

The central linear nucleus of the raphe had a dense distribution of AR‐IR nuclei (Figure 3: P3.2). A less dense distribution was observed in the parabrachial nucleus. The motor trigeminal nucleus showed a sparse distribution of AR‐IR nuclei (Figure 3: P3.2). No other areas of the pons, such as the pontine micturition and continence centers, the facial nucleus and areas of the cerebellum contained AR‐IR nuclei (Figure 3: P3.2, P7.5, Figure 4).

A high intensity of nuclear staining was observed in the central linear nucleus of the raphe. A slightly lower intensity of AR‐IR staining was observed in the parabrachial nucleus. The intensity of AR‐IR labeling was low in the motor trigeminal nucleus.

### 3.4 Mesencephalon

The distribution of AR‐IR nuclei was dense in the dorsal raphe nucleus, the PAG, the retrorubral nucleus, the substantia nigra (compact and reticular division), and the ventral tegmental area of Tsai (Figure 3: A6.1‐A0.6, Figure 4). The PAG showed numerous AR‐IR nuclei, throughout all its parts, except for the most central part directly adjacent to the ependymal lining, which did not contain AR‐IR nuclei (Figure 3: A6.1‐A0.6, Figure 4). Less dense labeling was found in the central tegmental field, the Edinger‐Westphal nucleus, the medial pretectal area, the mesencephalic tegmental field, and the superior colliculus (Figure 3: A6.1‐A0.6). A sparse distribution of AR‐IR nuclei was observed in the brachial nucleus of the inferior colliculus and the oculomotor nucleus (Figure 3: A6.1‐A0.6). AR‐IR nuclei were absent in the inferior colliculus, the red nucleus, and the trochlear nucleus (Figure 3: A4.1, A0.6).

Intensely stained nuclei were observed in the central tegmental field, the dorsal raphe nucleus, the Edinger Westphal nucleus, the mesencephalic tegmental field, the retrorubral nucleus, and the substantia nigra. The intensity of nuclear AR‐IR staining was less in the brachial nucleus of the colliculus inferior, the medial pretectal area, the oculomotor nucleus, the PAG, the superior colliculus, and the ventral tegmental area of Tsai.

## 4 DISCUSSION

This study provides the distribution of AR‐IR nuclei in the spinal cord and brainstem of the cat. The immunohistochemical results overlap with those of earlier studies in other species such as the rat. Here, we will focus on novel findings and on the overall significance of the distribution of AR‐IR nuclei. We firstly describe the similarities and differences with the distribution of androgen receptors as described in other species. Secondly, we discuss the involvement of the areas that contain AR‐IR nuclei in autonomic function and dysfunction. And thirdly, the distribution of androgen receptors in the cat is compared to that of estrogen receptors in the spinal cord and the brainstem.

### 4.1 Distribution of androgen receptors in the spinal cord and brainstem of the cat compared to that in other species

The density of AR‐IR nuclei in the spinal cord of the cat was moderate. A relatively dense distribution of AR‐IR nuclei was observed in the superior layers of the dorsal horn such as in layer I, in which primary afferent fibers terminate. Nuclei in the intermediate zone, lamina X, and the intermediolateral cell column were also AR‐IR. The present study shows that the intermediomedial cell column contains AR‐IR nuclei indicating that pre‐ganglionic sympathetic and parasympathetic motoneurons might express androgen receptors. These pre‐ganglionic motoneurons innervate the pelvic smooth musculature such as those in the bladder and bowel. Some of these AR‐IR areas at the lumbosacral level integrate visceral and somatic afferent and efferent information of the pelvic and pudendal nerves (Roppolo et al., 1985). The results obtained in the present study are consistent with those in studies in the rat (Lumbroso et al., 1996; Ranson et al., 2012). Studies investigating the expression of androgen receptors in the spinal cord of primates could not be found.

In the caudal brainstem, a dense distribution of AR‐IR nuclei was found in the area postrema, which is consistent with previous results in the rat (Hamson et al., 2004). The area postrema regulates various autonomic functions such as the control of respiration and blood pressure (Qian & Koon, 1998; Yang et al., 2006). The central linear nucleus of the raphe in the cat contained a dense distribution of AR‐IR nuclei, these neurons are serotonin producing neurons that project to the forebrain. The central linear nucleus is hypothesized to play a role in the sleep‐wake state of the organism (Trulson et al., 1981). AR‐IR nuclei were additionally observed in the dorsal motor vagus nucleus, consistent with previous results in the mouse (Mukudai et al., 2016; Yoon et al., 1996). The retrotrapezoid nucleus, a regulator of respiration automaticity, also contained a dense distribution of AR‐IR nuclei (Guyenet et al., 2019). The intensity of AR‐IR staining was little to absent in inferior olive, the motor nuclei of some of the cranial nerves (V, VII, IX, and XII), and the vestibular complex. The literature on androgen receptor expression in the motor nuclei of the cranial nerves in rats is conflicting. Hamson et al. did not find AR‐IR motoneurons in the dorsal vagus nucleus, the facial nucleus, the hypoglossal nucleus, and the motor trigeminal nucleus (Hamson et al., 2004). In contrast, another previous report in rats did show AR‐IR in these motor nuclei (Yu & McGinnis, 2001). Hamson et al. did show AR‐IR nuclei in the area postrema, the nucleus ambiguus, the parabrachial nucleus, the nucleus raphe magnus, and the nucleus of the solitary tract in the rat, which is consistent with the present study. In female rhesus monkeys, the uptake of dihydrotestosterone was studied and uptake was identified in the spinal trigeminal nucleus (Sheridan & Weaker, 1982). The present study in cats showed that AR‐IR nuclei in the spinal trigeminal nucleus are mainly located in the outer layer of the pars caudalis, an area that is involved in nociception (Patel & Das, 2019). Therefore, we can conclude that higher mammals such as cats and monkeys seem to have a similar distribution of androgen receptors in the motor nuclei of the cranial nerves.

In the mesencephalon, we observed a dense distribution of AR‐IR nuclei in the dorsal raphe nucleus in cats. The dorsal raphe nucleus is involved in the sleep‐wake state of the organism and it encodes reward signals (Li et al., 2016; McGinty & Harper, 1976). AR‐IR neurons in the dorsal raphe nucleus were previously described in male mice, rats and macaques (Bethea et al., 2015; Sheng et al., 2004). In the present study in cats, a high density of AR‐IR nuclei was observed in the periphery but not in the center of the PAG (Blok & Holstege, 1994; Faull et al., 2019). In rodents, AR‐IR neurons were mainly found in the dorsomedial and medial part of the PAG (Greco et al., 1996; Murphy et al., 1999; Simerly et al., 1990). The PAG regulates autonomic functions, such as cardiovascular control, micturition and respiration, in animals and humans. AR‐IR cells in the PAG might modulate autonomic behavioral responses such as sexual and urogenital reflexes that originate in the brainstem in both animals and humans (Blok et al., 1997; Marson & McKenna, 1996; Marson & Murphy, 2006; Michels et al., 2015). A dense distribution of AR‐IR nuclei in the male cat was also observed in the retrorubral nucleus, the substantia nigra, and the ventral tegmental area of Tsai, which plays a role in motivation and reward processing (Morales & Margolis, 2017). Similar results were obtained in the rat using immunohistochemistry, autoradiography, and in situ hybridization (Kritzer, 1997; Sar & Stumpf, 1975; Simerly et al., 1990). The substantia nigra in the cat contained AR‐IR nuclei in the reticular division as well as the compact division. In the rat, only the compact division showed AR‐IR cells (Kritzer, 1997). In conclusion, AR‐IR cells in the spinal cord, caudal brainstem, and the mesencephalon are highly conserved throughout species. A dense distribution of AR‐IR nuclei was present in regions that are involved in autonomic functions such as cardiovascular function, urogenital function, gastro‐intestinal function and respiratory function and in areas involved in processing of motivation and reward.

### 4.2 The involvement of androgen receptors in autonomic functions

Several areas with a high density of AR‐IR nuclei are involved in respiration. The areas that were identified in the present study are the area postrema, the dorsal motor vagus nucleus, the Kölliker‐Fuse nucleus, the nucleus of the solitary tract, the PAG, the parabrachial nucleus, and the retrotrapezoid nucleus. The area postrema has been implicated to alter cardiopulmonary responses in rats (Yang et al., 2006). Nerve fibers originating in the dorsal motor vagus nucleus innervate the muscles of the trachea and lower airways ensuring airway patency (Jordan, 2001). The Kölliker‐Fuse nucleus orchestrates the timing of the expiration phase (Barnett et al., 2018; Dutschmann et al., 2021). The PAG plays an important role in the integration of inputs regarding multiple autonomic functions (Faull et al., 2019). Furthermore, the parabrachial nucleus is thought to mediate respiratory rate (Miller et al., 2017). The retrotrapezoid nucleus is a regulator of breathing automaticity (Guyenet et al., 2019). The involvement of androgens is additionally supported by evidence that testosterone replacement in men with sleep apnea alters ventilation responses (Burschtin & Wang, 2016).

The areas involved in cardiovascular function that contained AR‐IR nuclei in the present study are: the area postrema, the dorsal motor vagus nucleus, the nucleus of the solitary tract, and the PAG. The area postrema and the nucleus ambiguus have been implicated to control heart rate (Gourine et al., 2016; Xue et al., 2003; Yang et al., 2006). The dorsal motor vagus nucleus controls the excitability of the ventricles of the heart (Gourine et al., 2016). Additionally, in rats it has been shown that the PAG is involved in the modulation of heart rate and blood pressure (Lagatta et al., 2016).

Furthermore, androgens play an important role in sexual function. Here, were described areas in the central nervous system that might be androgen sensitive indicated by a high density of AR‐IR nuclei. We showed a high density of AR‐IR nuclei in areas involved in sexual reflexes such as erection and orgasm. These areas include the PAG and the dorsal horn of the sacral spinal cord. The nucleus retroambiguus also contained AR‐IR nuclei. It has connections to the PAG and the lumbosacral spinal cord and is thought to be involved in mating behavior. The involvement of androgens in sexual function is supported by evidence that men with a decreased level of testosterone can experience a decrease in sexual desire and arousal, erectile dysfunction, reduced ejaculate, incontinence on orgasm, and reduced orgasm (Basaria et al., 2002; Casey et al., 2012; McGinty et al., 2014; Yeap, 2014). Decreased sexual desire has mostly been attributed to the effect of androgen deprivation in the telencephalon. However, this side effect can also possibly be attributed to an altered sensation of the external genitalia due to decreased androgen receptor activation in the dorsal horn of the sacral spinal cord. We found a relatively dense distribution of AR‐IR nuclei in the superficial layers of the dorsal horn, where sensory afferents from areas such as the genitals are located. Similarly, the effects could be mediated by other central nervous system areas with AR‐IR nuclei such as the PAG.

### 4.3 The distribution of androgen receptors compared to that of estrogen receptors

The distribution of estrogen receptors in the central nervous system is comparable between male and female rodents (Vanderhorst et al., 2005; Zhang et al., 2002). Also the androgen receptor distribution between male and female rodents is similar. However, it is not known whether the distributions of androgen and estrogen receptors overlap. A previous study described the estrogen receptor distribution in the spinal cord and brainstem of ovariectomized female cats. Remarkable overlap with the distribution of androgen receptors in the male cat exists. Estrogen receptors were detected in laminae I, II, V, VII, X, and in sacral pre‐ganglionic parasympathetic cells (VanderHorst et al., 2001). This is consistent with the distribution of androgen receptors in the male cat described in the present study. In the caudal brainstem of the female cat, estrogen receptors were present in the area postrema, the parabrachial nucleus, the solitary tract, and the outer layers of the pars caudalis of the spinal trigeminal nucleus (Boers, 2005). In the mesencephalon, estrogen receptors were observed in the brachial nucleus of the colliculus inferior, the dorsal raphe, the PAG, the superior colliculus, and the tegmentum (Boers, 2005). In ovariectomized female rhesus monkeys, a partly overlapping distribution of estrogen receptors was found (Vanderhorst et al., 2009). Estrogen receptors were present in laminae I‐V and X of the spinal cord, the pars caudalis of the spinal trigeminal nucleus, the solitary tract and the tegmentum in the caudal brainstem. In the mesencephalon, estrogen receptors were observed in the PAG and the parabrachial nucleus (Vanderhorst et al., 2009). Despite the great overlap between the estrogen receptor distribution in the cat and the monkey, estrogen receptor expression in the monkey was less abundant and less widespread than that in the cat. Whether this pattern is also observed in the distribution of androgen receptors in male primates is not known.

## 5 CONCLUSION

In this study, we showed specific areas in the spinal cord and brainstem of the cat in which AR‐IR nuclei are present. In the spinal cord of the cat the most dense distribution of AR‐IR nuclei was observed in the dorsal horn, which is involved in sensory processing. However, androgen receptor expression was more pronounced in supraspinal areas. AR‐IR nuclei in the brainstem of the cat were most prominent in the area postrema, the central linear nucleus, the dorsal motor vagus nucleus, the dorsal raphe nucleus, the PAG, the retrorubral nucleus, the retrotrapezoid nucleus, the substantia nigra, and the ventral tegmental area of Tsai. These areas are important for various autonomic functions such as cardiovascular function, micturition, respiration, and sexual function. These results underline the importance of androgen receptors in the central nervous system and their possible roles in sensory and autonomic functions. For clinical practice this means that patients with an altered availability of androgens or their receptors as is the case in hypogonadism, might experience autonomic dysfunction due to a decreased activation of androgen receptors in the spinal cord and brainstem.

## CONFLICT OF INTEREST

The authors declare no conflicts of interest.

## AUTHORS' CONTRIBUTIONS

RC contributed to data analysis/interpretation, drafting of the manuscript, and critical revision of the manuscript. JCC contributed to acquisition of data, data analysis/interpretation, and critical revision of the manuscript. PS and EA contributed to data analysis/interpretation and critical revision of the manuscript. BFMB contributed to the concept and design of the study, acquisition of data, data analysis/interpretation, and critical revision of the manuscript.

## Data Availability

Data available on request from the authors.
